# Factors Associated With the Vaccination Behavior Among COVID-19 Vaccine Hesitant College Students in Wuhan, China: A Survey Based on Social Psychological Dimension

**DOI:** 10.3389/fpubh.2022.865571

**Published:** 2022-05-11

**Authors:** Yifan Xiong, Ying Zhao, Tianyu Zhang, Qi Wang, Jun-an Liu

**Affiliations:** ^1^Department of Social Medicine and Health Management, School of Public Health, Tongji Medical College, Huazhong University of Science and Technology, Wuhan, China; ^2^Department of Social Medicine and Health Service Management, School of Public Health, Hengyang Medical School, University of South China, Hengyang, China; ^3^School of Basic Medicine, Tongji Medical College, Huazhong University of Science and Technology, Wuhan, China; ^4^Department of Epidemiology and Biostatistics, School of Public Health, Tongji Medical College, Huazhong University of Science and Technology, Wuhan, China

**Keywords:** vaccine hesitancy, college students, COVID-19 vaccine, psychological factors, vaccination behavior

## Abstract

**Background:**

During the COVID-19 pandemic, vaccine hesitancy (VH) on COVID-19 vaccination still exists in different populations, which has a negative impact on epidemic prevention and control. The objectives were to explore college students' willingness to vaccinate, determine the factors influencing the vaccination behavior of students with COVID-19 vaccine hesitancy, and provide a basis for improving the compliance of college students with COVID-19 vaccination.

**Methods:**

The universities in Wuhan are categorized into three levels according to their comprehensive strength and randomly sampled at each level, of which ten universities were selected. A self-designed anonymous electronic questionnaire was distributed online from May 12 to 31, 2021 to investigate the hesitancy, vaccination status, and influencing factors of COVID-19 vaccination among college students in Wuhan.

**Results:**

Of the 1,617 participants (1,825 students received the electronic questionnaire) surveyed, 19.0% reported COVID-19 vaccine hesitancy. Among the vaccine-hesitant students, 40.1% were vaccinated against COVID-19. The binary logistic regression analysis shows that families' attitudes “Uncertain” (odds ratio (OR) = 0.258 [0.132–0.503]), vaccination risk psychology (OR = 0.242 [0.079–0.747]) and wait-and-see mentality (OR = 0.171 [0.068–0.468]) are negative factors for the vaccination behavior of hesitant students, while herd mentality (OR = 7.512 [2.718–20.767]) and uncertainty of free policy's impact on vaccine trust (OR = 3.412 [1.547–7.527]) are positive factors.

**Conclusion:**

The vaccine hesitancy among college students in Wuhan was relatively high. Family support, herd mentality and free vaccination strategies can help improve vaccination among hesitant students, while vaccination risk psychology and “wait-and-see” psychology reduce the possibility of vaccination. The vaccination strategy of college students should be strengthened from the perspective of social psychological construction.

## Introduction

The novel coronavirus can invade the human body and cause severe respiratory diseases which may cause death. The COVID-19 pandemic has not been effectively controlled. As of January 16, 2022, 318 million people worldwide were infected with COVID-19 and more than 5.51 million died (1). The COVID-19 pandemic is currently considered as a major public health event by the World Health Organization (WHO) (2).

Vaccination is one of the most cost-effective strategies for COVID-19 infection prevention and population immunization (3, 4). Researches suggested that more than 70% of residents must be vaccinated to achieve population immunization (5). Judging from the current vaccination situation of the COVID-19 vaccines recommended by the WHO, COVID-19 vaccines are relatively safe and can effectively prevent COVID-19. Most people can get the COVID-19 vaccine without worrying about vaccination-associated severe adverse events (6). However, vaccine hesitancy (VH) persists as countries around the world vigorously promote COVID-19 vaccines (7–12). The WHO defines VH as a “delay in acceptance or refusal of vaccination despite availability of vaccination services” (13), which was listed as one of the ten threats to global health in 2019 (3). The incidence of VH varies among different populations. According to a survey in Europe, the vaccine hesitancy rate among Europeans is about 26.1% (9). Japan's survey shows that 11.3% of the population has VH (10). The vaccine hesitancy rate in the United States is about 35.4%, and the vaccine hesitancy rate in low- and middle-income countries in Asia, Africa, and South America is about 19.7% (11). China's surveys show that the hesitant rate of COVID-19 vaccines among domestic residents is about 8.4–35.5% (7, 14, 15). The emergence of VH threatens the completion of population immunization targets.

Existing research reports that the influencing factors of VH may include gender, knowledge background, perceived risk of COVID-19, cognition of vaccine, trust in vaccine, etc. (16), and vary among different populations. For example, the influencing factors of healthcare workers include vaccine safety, vaccine effectiveness, and distrust in the government (17). The influencing factors of dental students include economic background, gender, social media, public figures, insufficient knowledge about vaccines, and mistrust of governments and the pharmaceutical industry (18). Even though relevant studies supported the viewpoint that psychological factors such as distrust have an important impact on VH, there is still a lack of in-depth research on the impact of social psychological factors on VH, not to mention that the research on college students is quite limited. It is necessary to investigate the influence of social psychological factors on vaccine hesitancy in the college student population.

The National Health Commission of China stipulates that all adults voluntarily get the COVID-19 vaccination free of charge under informed consent. Wuhan, China launched the COVID-19 vaccination in all generally healthy adults (eligible for a COVID-19 vaccination) in March 2021. As of May 2021, Wuhan residents have been vaccinated with more than 9 million doses of COVID-19 vaccines. College students are one of the key protection groups for COVID-19 vaccination. Although all college students have the right to receive the free COVID-19 vaccination service, the emergence of vaccine hesitancy may reduce the coverage of the COVID-19 vaccine among college students. To improve the rate of COVID-19 vaccination, individuals' vaccination willingness should be improved to eliminate VH. The willingness of college students to vaccinate may be different from that of the ordinary adults, because the college student population is relatively immature and requires support from society and families, which means that the factors influencing college students' vaccination may be different from other populations. We hypothesized that social psychological factors play an important role in the occurrence of vaccine hesitancy among college students and could influence their vaccination behavior. Therefore, from a social psychology's perspective, this research aims to analyze the basic characteristics of college students with COVID-19 VH in Wuhan and explore the factors affecting the vaccination behavior of college students with VH to provide a scientific basis for improving the COVID-19 vaccination strategy for college students.

## Methods

### Research Design

#### Sample Selection

College students in Wuhan are the subjects of this study. The inclusion criteria of the research subjects are: (1) undergraduate students from different grades and majors in Wuhan; (2) college students who were willing and able to participate in the questionnaire survey. The exclusion criteria are: (1) graduate students and international students; (2) college students who refused or were unable to participate in the questionnaire survey.

With reference to QS World University Rankings 2022 and Shanghai Ranking's 2021 Best Chinese Universities Ranking (19, 20), colleges and universities in Wuhan are categorized into three levels according to their comprehensive strength (e.g., teaching level, scientific research level, personnel training, social influence, and other factors). The first level includes universities with strong comprehensive strength, the second level includes universities with medium comprehensive strength, and the third level includes universities with weak comprehensive strength. In all three levels of universities, random sampling was conducted with schools as the unit (two to three universities were randomly selected in the first and second levels of universities. Given that many universities are in the third category, universities are continuously divided into two types according to the existence of medical specialties and two to three universities were randomly selected in each level).

The study eventually selected two first-level universities (Wuhan University and Huazhong University of Science and Technology), two second-level universities [Wuhan University of Technology and China University of Geosciences (Wuhan)], and six third-level universities (i.e., three universities with medical specialties and three universities without medical specialties) (Wuhan Engineering University, South-Central University for Nationalities, Wuhan Textile University; Wuhan University of Science and Technology; Jianghan University and Hubei University of Traditional Chinese Medicine). Ten universities were selected for this study. The majors of the students that were collected in the questionnaire were divided into four categories: engineering (e.g., machinery, automation), science (e.g., physics), medicine (e.g., clinical medicine, dental, pharmaceutical pharmaceutics, preventive medicine, nursing, and other healthcare students), and humanities and social sciences (e.g., sociology, literature).

According to the formula of sample size required for the cross-sectional survey, $n=p\left(1-p\right){\left(\frac{Z_{1-\frac{\alpha }{2}}}{\delta }\right)}^{2}$. The *p*-value was estimated by the influenza vaccination rate of Chinese adults during the influenza epidemic period from 2009 to 2010 (21), *p* ≈ 51.5%, and the explicit level α = 0.05, and the absolute tolerance error δ = 5%. This study is a stratified cluster random sampling survey, and the Deff (design effect) = 2. Taking into account the 20% non-response rate, the minimum sample size required was calculated as *n* = 921. During the survey, the electronic questionnaire was distributed to 1,825 people, and a total of 1,712 people participated in the questionnaire survey, with a response rate of 93.81%. At last, 1,617 valid questionnaires were collected, with an effective rate of 94.45%. The sample size in this study fully met the research requirements in accordance to the sampling survey formula, which meant our study was representative of a certain extent.

#### Investigation Process

The online survey was conducted from May 12 to May 31, 2021. Questionnaire design, distribution, and data collection were completed on the Wen Juan Xing platform (Changsha Ranxing Information Technology Co., Ltd., Hunan, China) which is one of the largest online survey platforms in China. 1) The researchers completed the design of electronic questionnaires and generated QR codes and hyperlinks through the Wen Juan Xing platform. 2) Fifty classes from the 10 selected universities were randomly selected. The link and QR code of the electronic questionnaire were distributed through the QQ group of the selected class (a social networking platform used by students in China for daily communication, similar to Facebook's chat group). 3) Students voluntarily fill out the questionnaire through the online platform with informed consent. For QQ groups with low response rates, the investigator will issue reminders during students' free time to increase the response rate. The electronic questionnaire was submitted only after all answers were given. 4) The data of the respondents will be automatically uploaded and saved to the Wen Juan Xing platform, and the researchers can download and analyze the data of the participants.

#### Quality Control

1) Before filling out the questionnaire, all participants read the questionnaire instructions and understood the precautions for filling in the questionnaire.
2) During the completion of the electronic questionnaire, participants can freely seek help from the investigator to solve their doubts in the questionnaire filling.
3) Each participant who completed the questionnaire was rewarded with CNY 2-5 randomly through Wen Juan Xing platform to encourage the completion of the questionnaire.
4) The electronic questionnaire was also set with IP address restrictions and the same computer/mobile phone restriction to prevent repeated responses.
5) The study was approved by the Ethics Committee of Tongji Medical College, Huazhong University of Science and Technology [2021-S214]. The anonymous questionnaire was filled out voluntarily with the participants' informed consent.

### Measures

#### Theoretical Basis

The determination of vaccine hesitancy in the questionnaire refers to the WHO's definition of vaccine hesitancy (13). According to the vaccine hesitancy 3C model provided by the WHO, the model classifies the influencing factors of vaccination into three categories: confidence, complacency, and convenience (3C) (13). This model has strong reliability and validity in explaining vaccine hesitancy. Based on this, this study measures the psychological dimensions of college students' herd psychology, wait-and-see attitude, vaccination risk psychology, and free vaccination's impact to explore the influence of social psychological factors on vaccination behavior of vaccine-hesitant people.

#### Measuring Tools

After consulting the relevant literature and experts, a questionnaire was compiled according to the research purpose. The content of our questionnaire includes the sociodemographic characteristics of the respondents, psychological factors (e.g., herd mentality, wait-and-see mentality, vaccine risk psychology, and price psychology), vaccination willingness, and vaccination behavior.

#### Measurement of Psychological Factors

Items of psychological problems in this study include: [1] Do you have a herd mentality? [2] Do you have a wait-and-see mentality? [3] Do you think the risks of vaccination outweigh its benefits? [4] Will the free vaccine affect your trust in it? The answer to the question is designed as a Likert five-point scale, from 1 to 5, representing “Not at all, Slightly, Intermediate, Yes, probably, Yes, absolutely.” Each psychological factor was selected and measured by the interviewees in the aforementioned manner.

In the data analysis process, for the combination with the psychological factor options, the participants who choose “Not at all” and “slightly” belong to “No,” while participants who choose “Yes, probably” and “Yes, absolutely” belong to “Yes.” Participants who select the option “intermediate” are classified as “Uncertain.”

#### Assessment of Vaccine Hesitancy

The questionnaire asks the following: “Would you like to get the novel coronavirus vaccine?”. The answer to this question can be either of the following five options: absolutely unwilling, probably unwilling, intermediate, probably willing, or absolutely willing. According to the WHO's definition of VH, students who choose “absolutely unwilling,” “probably unwilling,” and “intermediate” are classified as vaccine hesitators. Students who choose “probably willing” and “absolutely willing” belong to vaccine acceptors.

### Statistical Analysis

In this study, Wen Juan Xing was used to build a database, and SPSS version 26 (IBM Corporation, New York, NY, USA) was used for data analysis. The χ^2^ test was used to investigate the influencing factors of 1,617 surveyed students' vaccine hesitancy and compare the differences in sociodemographic characteristics and psychological factors between 307 vaccinated and unvaccinated hesitant students, and the influencing factors with statistical significance were screened out.

In order to identify the influencing factors of hesitant students' vaccination behavior and eliminate the influence of confounding factors, we applied a stepwise binary logistic regression analysis (The parameter estimation method of the model is maximum-likelihood estimation, MLE; the parameter testing method is wald test; the testing level is α>0.05). The COVID-19 vaccination behavior was used as the dependent variable (“vaccinated” and “unvaccinated” were assigned at 1 and 0, respectively), and items such as gender, grade, university level, monthly living expenses, major, family attitude and psychological factors were used as independent variables. Categorical variables were treated as dummy variables in the analysis. The Hosmer-Lemeshow test was used to determine the applicability of the multivariate analysis model at a test standard of *P* > 0.05.

## Results

### Sociodemographic Characteristics of 1,617 Participants

**Figure 1:**
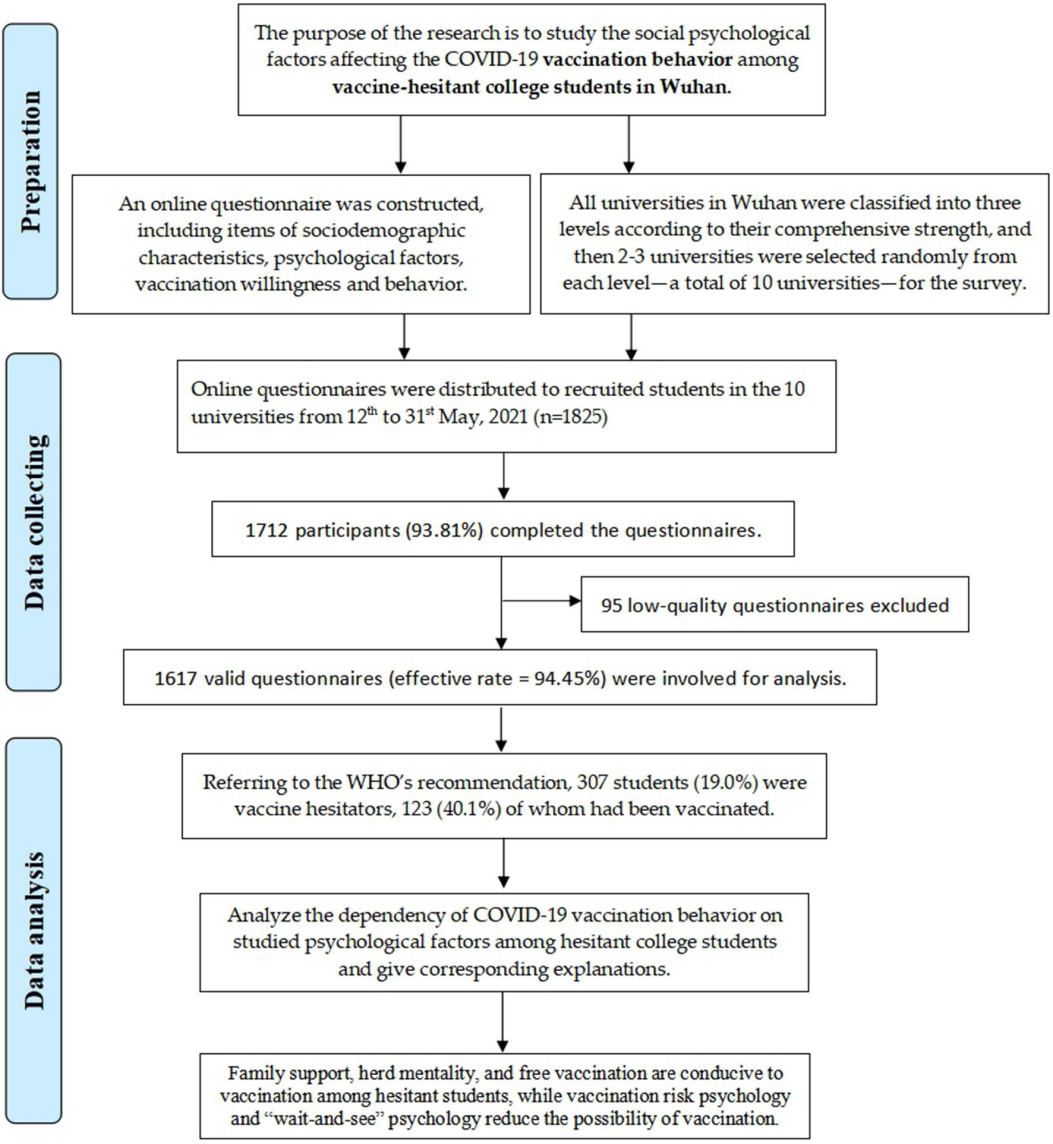
Flow diagram for the study outline.

The study outline of this research is shown in Figure 1. Among the 1,617 involved college students, 729 (45.1%) were male. Total of 530, 371 and 716 students were from the first-, second-, and third-level schools, respectively. The monthly living expenses of most students (*n* = 781) were about CNY 1,000–1,500; 161 students were native in Wuhan; and the participants were of diverse grade and major distributions. Table 1 describes the detailed characteristics and vaccination status (against COVID-19) of the surveyed students.

**Table 1:** Characteristics and vaccination status of participants (*n* = 1,617).

**Characteristics**	***n***	**Vaccine acceptant (*****n** **=*** **1,310)**		**Vaccine hesitant (*****n** **=*** **307)**		***p***
		***N***	**%**	***N***	**%**	
**Gender**						<0.001^***^
Male	729	556	76.3	173	23.7	
Female	888	754	84.9	134	15.1	
**University Level**						0.012^*^
First kind	530	413	77.9	117	22.1	
Second kind	371	294	79.2	77	20.8	
Third kind	716	603	84.2	113	15.8	
**Monthly cost of living**						0.588
< CNY 1,000	217	169	77.9	48	22.1	
CNY 1,000–1,500	781	644	82.5	137	17.5	
CNY 1,500–2,000	474	380	80.2	94	19.8	
>CNY 2,000	145	117	80.7	28	19.3	
**Years of study**						0.077
One	494	395	80.0	99	20.0	
Two	505	395	78.2	110	21.8	
Three	398	335	84.2	63	22.0	
Four and more	220	185	84.1	35	15.9	
**Major**						0.060
Engineering	670	523	78.1	147	21.9	
Science	257	213	82.9	44	17.1	
Medicine	266	226	85.0	40	15.0	
Humanities and Social sciences	424	348	82.1	76	17.9	
**Native place**						0.049^*^
Wuhan, Hubei Province	161	127	78.9	34	21.1	
Other cities in Hubei Province	108	96	88.9	12	11.1	
Used to be medium-high-risk areas outside Hubei Province	869	689	79.3	180	20.7	
low-risk areas outside Hubei Province	479	398	83.1	81	16.9	
**Vaccination status**						<0.001^***^
Vaccinated	1,232	1,109	84.7	123	40.1	
Unvaccinated	385	201	15.3	184	59.9	

* *p < 0.05*;

*** *p < 0.001*.

Of the 1,617 participants, 1,232 (76.2%) had been vaccinated against COVID-19, and 307 (19.0%) were vaccine-hesitant students. And not surprisingly, the vaccine acceptant individuals had significantly higher vaccination proportion (84.7%) than the vaccine hesitant ones (40.1%) (*p* < 0.001). Referring to the vaccination status, those vaccine-hesitant students can be classified into two categories: unvaccinated hesitators (*n* = 184, 59.9%) and vaccinated hesitators (*n* = 123, 40.1%).

### Differences in Vaccination Status Among COVID-19 Vaccine Hesitant Students

Table 2 summarizes the differences in vaccination status among the COVID-19 vaccine hesitant students with different characteristics. Among the 307 vaccine-hesitant college students, 173 (56.4%) were male; and 117 (38.1%), 77 (25.1%), and 113 (36.8%) students were from the first-, second-, and third-level universities, respectively. A total of 34 (11.1%), 81 (26.4%), 12 (3.9%), and 180 (58.6%) students were native from Wuhan, other cities in Hubei Province, medium-high-risk areas outside Hubei Province, and low-risk areas outside Hubei Province, respectively. The COVID-19 vaccine hesitant college students in the survey were distributed in all majors and grades.

**Table 2:** Comparison of vaccination status among the COVID-19 vaccine hesitators with different characteristics.

**Characteristics**	***n***	**Vaccinated (*****n** **=*** **123)**		**Unvaccinated (*****n** **=*** **184)**		***p***
		***n***	**%**	***n***	**%**	
**Gender**						0.212
Male	173	64	37.0	109	63.0	
Female	134	59	44.0	75	56.0	
**School Level**						<0.001^***^
First kind	117	29	24.8	88	75.2	
Second kind	77	43	55.8	34	44.2	
Third kind	113	51	45.1	62	54.9	
**School Type**						0.040^*^
Medical college	21	12	57.1	9	42.9	
Comprehensive University with Medicine	157	53	33.9	104	66.2	
Comprehensive universities without Medicine	129	58	45.0	71	55.0	
**Years of study**						0.017^*^
One	99	42	42.4	57	57.6	
Two	110	32	29.1	78	70.9	
Three	63	33	52.4	30	47.6	
Four and more	35	16	45.7	19	54.3	
**Major**						0.182
Engineering	147	50	34.0	97	66.0	
Science	44	19	43.2	25	56.8	
Medicine	40	17	42.5	23	57.5	
Humanities and Social Sciences	76	37	48.7	39	51.3	
**Classification of Specialties**						0.736
Medical specialty	40	23	57.5	17	42.5	
Non-medical specialty	267	161	60.3	106	39.7	
**Monthly cost of living**						0.069
< CNY 1,000	48	18	37.5	30	62.5	
CNY 1,000–1,500	137	61	44.5	76	55.5	
CNY 1,500–2,000	94	39	41.5	55	58.5	
>CNY 2,000	28	5	17.9	23	82.1	
**Have any of your relatives studied medicine?**						0.127
Yes	95	32	33.7	63	66.3	
No	212	91	42.9	121	57.1	
**Native place**						0.568
Wuhan, Hubei Province	34	11	32.4	23	67.6	
Other cities in Hubei Province	81	37	45.7	44	45.7	
Used to be medium-high-risk areas outside Hubei Province	12	5	41.7	7	58.3	
low-risk areas outside Hubei Province	180	70	38.9	110	61.1	
**Do your family support your vaccination?**						<0.001^***^
Support	200	104	52.0	96	48.0	
Unclear	98	16	16.3	82	83.7	
Oppose	9	3	33.3	6	66.7	

* *p < 0.05*;

*** *p < 0.001*.

χ^2^ test is used to compare the differences in vaccination behavior of VH students with different characteristics. The results of χ^2^ test show significant differences in vaccination behavior among students with different university levels, university types, grades, and family attitudes toward vaccination (*p* < 0.05). Gender, major, native place, whether nucleic acid testing has been done, whether family members or acquaintances have been infected with COVID-19, and the vaccination status of family members or acquaintances have no effect on the vaccination behavior of VH students (*p* > 0.05, some non-significant factors are not shown in the table).

### Psychological Factors Analysis Among Vaccine Hesitant Students

The COVID-19 vaccine hesitators with wait-and-see and vaccination risk psychology, and without herd psychology were less likely to be vaccinated; and the COVID-19 vaccine hesitators with “unsure” attitude about free vaccines on trust were more likely to be vaccinated (Table 3).

**Table 3:** Effect of psychological factors on vaccination behavior of COVID-19 vaccine hesitators.

**Items**	***n***	**Vaccinated (*****n** **=*** **123)**		**Unvaccinated (*****n** **=*** **184)**		***p***
		***n***	**%**	***n***	**%**	
**Do you have a herd mentality?**						<0.001^***^
No	97	22	22.7	75	77.3	
Intermedia/unsure	163	76	46.6	87	53.4	
Yes	47	25	53.2	22	46.8	
**Do you have a wait-and-see mentality?**						<0.001^***^
No	59	28	47.5	31	52.5	
Intermedia/unsure	143	74	51.7	69	48.3	
Yes	105	21	20.0	84	80.0	
**Do you think the risks of vaccination outweigh its benefits?**						<0.001^***^
No	84	35	41.7	49	58.3	
Intermedia/unsure	170	80	47.1	90	52.9	
Yes	53	8	15.1	45	84.9	
**Will the free vaccine affect your trust in it?**						<0.001^***^
No	136	34	25.0	102	75.0	
Intermedia/unsure	149	82	55.0	67	45.0	
Yes	22	7	31.8	15	68.2	

*** *p < 0.001*.

### Logistic Regression Analysis of Vaccination Behavior of Vaccine Hesitant Students

In this research, the vaccination behaviors of vaccine-hesitant students were used as the dependent variable (“vaccinated” and “unvaccinated” were assigned at 1 and 0, respectively), while gender, university type, grade, family attitude, herd mentality, wait-and-see mentality, vaccination risk psychology, and free vaccination's influence were used as independent variables. Binary logistic regression analysis (Table 4) showed that the factors related to the vaccination behavior of vaccine-hesitant college students included family attitude toward students' vaccination, herd mentality, wait-and-see mentality, free vaccination's influence, and the assessment of risks and benefits of vaccination. Family attitudes “Uncertain” [odds ratio (OR) = 0.258, 95% CI = 0.132–0.503], vaccination risk psychology (OR = 0.242 95% CI = 0.079–0.747) and wait-and-see mentality (OR = 0.171, 95% CI = 0.068–0.468) are negative factors for vaccine hesitant students' vaccination behavior, while herd mentality (OR = 7.512, 95% CI =2.718–20.767) and uncertainty of the impact of free policy on vaccine trust (OR = 3.412, 95% CI = 1.547–7.527) are positive factors for vaccine hesitant students' vaccination behavior.

**Table 4:** Factors associated with vaccination behaviors among the COVID-19 vaccine hesitators.

**Items**	**β**	**OR**	**95% CI**	***p***
**Do your family support your vaccination?**
Support		Ref	Ref	
Unclear	−1.355	0.258	0.132–0.503	<0.001^***^
Oppose	−0.835	0.434	0.078–2.412	0.340
**Do you have a herd mentality?**
No		Ref	Ref	
Unsure	0.985	2.677	1.201~5.970	0.016^*^
Yes	2.017	7.512	2.718~20.767	<0.001^***^
**Do you have a wait-and-see mentality?**
No		Ref	Ref	
Unsure	−0.879	0.415	0.171–1.008	0.052
Yes	−1.722	0.171	0.068–0.468	<0.001^***^
**Do you think the risks of vaccination outweigh its benefits?**
No		Ref	Ref	
Unsure	−0.739	0.482	0.212–1.097	0.082
Yes	−1.418	0.242	0.079–0.747	0.014^*^
**Will the free vaccine affect your trust in it?**
No		Ref	Ref	
Unsure	1.227	3.412	1.547~7.527	0.002^**^
Yes	1.118	3.059	0.853~10.965	0.086

*In the table: the second column: β is the maximum likelihood estimate of the regression coefficient; the third column is the estimated value of the odds ratio (OR); the fourth column is the upper and lower limits of the 95% confidence interval of the odds ratio OR; the fifth column is the P-value of the Wald χ^2^ test*;

* *p < 0.05*;

** *p < 0.01*;

*** *p < 0.001*.

## Discussion

The results show that college students in Wuhan can be vaccinated against COVID-19 with a VH rate of 19.0%, which is lower than that of French students (42.0%), and higher than that of Italian students (13.9%) (22, 23). Compared with a vaccine hesitancy rate of 8.4% in a large-scale national study in China, college students in Wuhan showed a higher vaccine hesitancy rate (15). In this study, we found that 184 (59.9%) of the 307 vaccine-hesitant students had not yet been vaccinated. These data are much higher than the overall non-vaccination rate of surveyed students (23.8%), which shows that vaccine hesitancy can significantly reduce students' enthusiasm for vaccination. Of the 307 vaccine-hesitant students, 123 (40.1%) were vaccinated, indicating that vaccine hesitators are likely to be vaccinated under the influence of certain factors. Existing studies have found that psychological factors play an important role in perceiving VH (24–27). This study found that the social psychological factors, including family's attitude toward child's vaccination, herd mentality, wait-and-see mentality, free vaccination's impact, and thinking the risk of vaccination is greater than its benefit, had an impact on vaccination behaviors among hesitant college students.

Family attitudes are essential to college students' willingness to vaccinate against COVID-19. In comparison with family support attitudes, students with unclear family attitudes were less likely to vaccinate with the COVID-19 vaccine. A study in Jordan found that only 30.2% of parents were willing to vaccinate their children against COVID-19 (28). Family support is an important factor affecting health behavior choices, especially from parents' suggestions. Studies have shown that family attitudes toward vaccination directly affect college students' vaccination psychology, thus affecting college students' vaccination behaviors (29, 30). Therefore, the vaccination rate of hesitant students can be increased by changing parents' attitudes toward vaccination.

Herd mentality has a significant positive effect on vaccination for college students with COVID-19 VH. The research demonstrated a higher likelihood of vaccination behavior in vaccine-hesitant students who chose “Yes” or “Uncertain” for their assessment for herd mentality. Students can increase their confidence in the vaccine from other people's vaccination behavior, and students' vaccination behavior is also driven by positive valence related to social consistency, from which an individual can obtain positive valence such as social identity and superiority through behaving consistently with other people (31). Students are considered as trusted influencers and ambassadors for vaccine promotion, and advice from healthcare providers and encouragement from close ones (i.e., parents and peers) are also critical to vaccination decisions (32). Therefore, the vaccination rate can be improved by influencing herd pressure, such as expert recommendations, publicity coverage expansion and the demonstration effect of the surrounding vaccinated individuals.

The wait-and-see mentality originates from concerns about the uncertainty of the vaccine, which appears in most people with VH (33–35). The results of this study showed that wait-and-see mentality may be a negative influencing factor of vaccination, with an OR value of 0.171 (0.068–0.468). A wait-and-see attitude is associated with the risk of infection. College students think that they are not easy to get infected and their perceived risk of COVID-19 is weak, which is likely to result in a wait-and-see attitude (35). Wait-and-see attitudes may arise from the distrust of vaccines. Propagandizing the safety of vaccines by early vaccinated people helps reduce distrust in vaccine safety. Therefore, it is recommended that relevant departments collect and analyze data on the side effects and protective effects of vaccination and publish them in a timely manner. Scientific data should be publicly promoted in time to eliminate the wait-and-see attitude of vaccine hesitators and change their vaccination behavior.

The epidemiological and social crises brought about by COVID-19 have magnified widely held social anxieties and trust issues that, in the unique circumstances of this global pandemic, have exacerbated skepticism toward vaccines. Trust is key to overcoming vaccine hesitancy (36). Since vaccination is a kind of health protection behavior, vaccine-hesitant college students who believe that the risks of vaccination outweigh its benefits are less likely to be vaccinated with an OR value of 0.242 (0.079–0.747) because of their distrust in vaccines. In the VUCA model, COVID-19 is a new infectious disease, which is more complicated and uncertain (37). People are easily affected by misinformation and adopt irrational behaviors. Given the short development period of COVID-19 vaccines, coupled with the vaccination accidents of related vaccines, poor vaccine protective efficacy, and other facts, college students' willingness to vaccinate is easily affected by negative news about COVID-19 vaccination (38). Studies have found that the overall quality and credibility of the information about the COVID-19 vaccine on the famous video sharing platform YouTube is poor, and the false information contained therein may reduce people's COVID-19 vaccination rate (39). Jain et al. suggest that it is pertinent to design an evidence-based strategy to promote the uptake of vaccination among students, which could include informational campaigns that address vaccine hesitancy (40). Therefore, the government needs to strengthen the popularization of vaccine knowledge and correcting misinformation to improve people's trust in the vaccine, thereby improving the hesitators' willingness to vaccinate (41, 42). Based on the characteristics of college students, typical approaches include lectures, campus posters, and social media promotion can be used to increase college students' awareness of vaccines and increase their enthusiasm for vaccination.

Economic research shows that the price of goods affects customers' judgment of their quality, thereby affecting their purchase behavior (43). In general, most people are skeptical about free things, but free vaccines can increase the availability of vaccination, thus encouraging people to participate in vaccination. Related studies have shown that healthy college students have a low level of disease risk perception and are prone to complacency for health status (24). According to the expectancy-value theory (44), people may think items that are too easy to obtain are of poor quality and limited effects, therefore attenuating the perceived value. Similarly, the free vaccine policy may reduce people's enthusiasm for vaccination. However, the cost of the vaccine is another reason for people's unwillingness to vaccinate (45). This ambivalence may also be a deep-seated cause of vaccine hesitancy. Because China's COVID-19 vaccine is free, this study set up the question “Will the free vaccine affect your trust in it?”. However, only 7.17% of students thought it had a negative impact on vaccine trust, and most students did not reduce their confidence in vaccines because of the free vaccination policy. Logistic regression analysis results show that people who are uncertain about whether the free vaccine policy affects trust are more likely to be vaccinated. Therefore, a free vaccination strategy is necessary to prevent the reduction of vaccination rates due to economic factors.

This project was an earlier study aimed specifically at Chinese college students' COVID-19 vaccine hesitancy, vaccination status and influencing factors. The advantage of this research lies in analysis of influencing factors of hesitant college students' vaccination behavior from the social psychological dimension. The study found that the herd mentality effect can be strengthened to promote hesitant college students' vaccination. In order to increase the vaccination rate of hesitant students, the following policy implications can be obtained based on our research results: 1) Carry out peer education in class to increase the pressure of conformity, thereby promoting the vaccination of hesitant students. 2) Through the publicity of medical authorities, eliminate the concerns of college students about the uncertain factors of vaccines. 3) Improve the family's attitude toward vaccination through publicity to the hesitant students' families, thereby promoting students' enthusiasm for vaccination. 4) The government and media should manage the information about the COVID-19 vaccine on the Internet, promote the real information of the vaccine, and correct the false information in time.

However, there are still some limitations in our study. Firstly, the proportion of vaccine hesitators in the population is relatively small, which makes the number of hesitant students in our survey smaller, which may lead to statistical insignificance of some factors that affect vaccination. Secondly, the sample we surveyed includes non-responding students, which may cause selection bias. Lastly, the research is cross-sectional, and a lack of control group may have some confounding biases in the identification of influencing factors.

## Conclusion

The rate of COVID-19 VH among college students in Wuhan was 19.0%. Family support, herd mentality, and free vaccination are conducive to vaccination among hesitant students, while vaccination risk psychology and “wait-and-see” psychology reduce the possibility of vaccination. The vaccination strategy of college students should be strengthened from the perspective of social psychological construction.

## Data Availability Statement

The original contributions presented in the study are included in the article/supplementary material, further inquiries can be directed to the corresponding author/s.

## Ethics Statement

The studies involving human participants were reviewed and approved by the Ethics Committee of Tongji Medical College, Huazhong University of Science and Technology. The patients/participants provided their written informed consent to participate in this study.

## Author Contributions

YZ and JL designed the study. YX and TZ carried out the investigation. YX, YZ, and QW carried out the analysis and interpreted the results. YX drafted the manuscript. TZ, QW, and JL assisted in drafting and reviewing the manuscript. All authors read and approved the final manuscript.

## Funding

The work was funded by the National Natural Science Foundation of China [Grant No. 72061137006] and the Fundamental Research Funds for the Central Universities [Grant No. 2019kfyXKJC003].

## Conflict of Interest

The authors declare that the research was conducted in the absence of any commercial or financial relationships that could be construed as a potential conflict of interest.

## Publisher's Note

All claims expressed in this article are solely those of the authors and do not necessarily represent those of their affiliated organizations, or those of the publisher, the editors and the reviewers. Any product that may be evaluated in this article, or claim that may be made by its manufacturer, is not guaranteed or endorsed by the publisher.
